# Recognition of reviewers

**DOI:** 10.1210/jendso/bvag005

**Published:** 2026-03-11

The editors of *Journal of the Endocrine Society* thank all those who reviewed during 2025 for their time and effort.

Ali Abbara

Bo Abrahamsen

Safwaan Adam

Adegbenga Ademolu

Robert Adler

Sunita Agarwal

Manuel Aguiar-Oliveira

S. Faisal Ahmed

Benson Akingbemi

Dalal Ali

Carolyn Allan

Roberta Allgayer

Jeremy Allgrove

Laura Alonso

Ferdinand Althammer

Francisco Álvarez-Nava

Ali Alzahrani

Sayeepriyadarshini Anakk

Richard Anderson

Takao Ando

Stanley Andrisse

Trevor Angell

Younes Anini

Ansarullah Ansarullah

Zoltan Antal

Sonir Antonini

Takako Araki

Magnolia Ariza-Nieto

Decio Armanini

Andrew Arnold

Ambika Ashraf

Craig Atwood

Richard Auchus

Laura Audi

Renata Simona Auriemma

Ricardo Azziz

Vedesh Babu

Leon Bach

Tania Bachega

Philippe Backeljauw

Ramiro Balderas

Maria Paula Bandeira

Giuseppe Barbesino

Joshua Barzilay

Sarah Bath

Rafael Batista

Traci Bekelman

Scott Belcher

Margaret Bell

Denise Belsham

Jerzy Beltowski

Chris Benner

Darlene Berryman

Jerome Bertherat

Traci Bethea

Matthias Betz

Gerhard Binder

Bernadette Biondi

Jenny Bischoff

Robert Blank

Sandra Blethen

Bruce Blumberg

Richard Bockman

Anita Boelen

Jens Bollerslev

Kleiton Borges

Roger Bouillon

Alison Boyce

Matthew Brady

Joseph Braun

Gregory Brent

David Broome

Rebecca Brown

Thierry Brue

Gary Butler

Sally Camper

Irene Campi

Marina Caputo

Ruben Cardozo

Owen Carmichael

Thomas Carpenter

Fernando Cassorla

Frederic Castinetti

Jane Cauley

Melissa Cavaghan

Filippo Ceccato

Sourav Chakraborty

Raquel Chamorro-Garcia

Ching-yi Chang

Lily Chao

Debbie Chen

Ana Cheong

Ada Cheung

George Chrousos

Antonio Cittadini

Catherine Cohen

Laurie Cohen

Barbara Cohn

Paulo Collett-Solberg

Fabio Comim

Colin Conine

Robert Considine

Fernanda Correa

Sara J. Cromer

Silas Culver

Carolyn Cummins

Tim Cundy

Rebecca Cunningham

Mauro Czepielewski

Samuel Dagogo-Jack

Kevin Dalby

Adrian Daly

Matthew Dapas

Caroline Davidge-Pitts

Shanlee Davis

Bess Dawson-Hughes

Margaret de Castro

Miguel Debono

Priya Dedhia

Sara Della Torre

Francesco DeMayo

Arash Derakhshan

Kaniksha Desai

Mario Detomas

Ruban Dhaliwal

Waljit Dhillo

Beverly Diamond

Paola Divieti Pajevic

Chrysoula Dosiou

Daniel Drucker

Daisy Duan

Quan-Yang Duh

Daniel Dumesic

Nancy Dunbar

Grigoris Effraimidis

Dariush Elahi

Ghada El-Hajj Fuleihan

Joel Elmquist

Maria Escobar-Vasco

Erica Eugster

Gustavo Fagundes

Thomas Fahey III

Andrea Ferreira

Jonathan Flak

Maria Fleseriu

Lindsay Fourman

Maria Candida Fragoso

Patrick Fueger

Maki Fukami

John Fuqua

Mônica Gadelha

Eliza Geer

William Gibbons

Andrea Giustina

Andrea Glezer

Celso Gomez-Sanchez

Elise Gomez-Sanchez

Andrea Gore

Daniel Gorelick

Caroline Gorvin

Haixia Guan

Heather Guetterman

Tulay Guran

Mouhammed Habra

David Halsall

Stephen Hammes

Ole-Petter Hamnvik

Amir Hamrahian

David Handelsman

Vincent Harley

Kylie Harrall

Martin Hewison

Emily Hilz

Ken Ho

Christine Hockett

Andrew Hoffman

Daniel Holmes

Elizabeth Holt

William Horton

Sandra Hunter

Lourdes Ibanez

Peter Igaz

Ioannis Ilias

Michael Irwig

Najmul Islam

Nobuaki Ito

Jarmo Jääskeläinen

Smita Jha

Gudmundur Johannsson

Erik Joly

Jens Otto Jørgensen

George Kahaly

Roopa Kanakatti Shankar

Paul Kaplowitz

Niki Karavitaki

Atil Kargi

Christopher Kassotis

Leandro Kasuki

Electron Kebebew

Bernard Khoo

Cari Kitahara

Gregory Kline

Christian Koch

Josef Köhrle

Marta Korbonits

Anupam Kotwal

Stephanie Kullmann

Manikya Kuriti

Deborah Kurrasch

Enzo Lalli

Nils Lambrecht

Inigo Landa

Bruce Lanphear

Neil Lawrence

Phillip Lee

Hang Lee

Svenja Leibnitz

Ellen Leschek

Jon Levine

Chien Li

Rong Li

Xia Li

Sarah Little-Letsinger

Chienying Liu

Jianmin Liu

Alejandro Lomniczi

Carlos Longui

Miguel López

Kaitlin Love

Xiaoping Luo

Mohamad Maghnie

Joseph Majzoub

Aristides Maniatis

Michael Mannstadt

Pedro Marques

George Mastorakos

Christopher McCartney

Shana McCormack

Per Medbøe Torsby

Onno Meijer

Philippa Melamed

Shlomo Melmed

Berenice Mendonca

Jan Mennigen

Moises Mercado

Livia Mermejo

Tyler Milewski

Bradley Miller

Alex Miras

Emilia Modolo Pinto

Paolo Moghetti

Gylli Mola

Arnaud Molin

Alina Montalbano

Mariana Monteiro

Yasaman Motlaghzadeh

Noel Mueller

Paolo Mulatero

Ranganath Muniyappa

Robert Murray

Madalina Musat

Kazutaka Nanba

Robert Nerenz

Hannah Nieto

Hiroshi Nishi

Natalie Nokoff

Jerome Nwachukwu

Ohn Nyunt

Onyebuchi Okosieme

James Olcese

Michael O'Reilly

Keiichi Ozono

Vasantha Padmanabhan

Ticiana Paes

Christina Pamporaki

Rosa Maria Paragliola

Kepal Patel

Rocio Pereira

Tommaso Piticchio

Rosario Pivonello

Melissa Premaor

Flavia Prodam

Stuart Ralston

Sudhaker Rao

Frank Rauch

Julie Refardt

Martin Reincke

Jun Ren

Rodolfo Rey

Megan Ritter

Marie-Eve Robinson

Cassianne Robinson-Cohen

Manuela Rocha-Braz

Troy Roepke

Alan Rogol

Lisa Rokoff

Allen Root

Pedro Rosario

Stephen Rosenthal

Benjamin Rösing

Janet Rubin

Paul Saenger

Yoshifumi Saisho

Marzieh Salehi

Domenico Salvatore

Roberto Salvatori

Mario Salvi

Robert Sargis

Sabitha Sasidharan Pillai

Tetsuhiko Sato

Carla Scaroni

Thomas Scherer

Arthur Schneider

Nadia Schoenmakers

Ann Schwartz

Jowy Yi Hoong Seah

Carolyn Seib

Sabyasachi Sen

Jad Sfeir

Joseph Shaker

Neil Sharma

Dana Sheinhaus

Angela Sheu

Paula Silva

Jennifer Sipos

Daniel Spratt

Poli Mara Spritzer

Brendan Stack

Takara Stanley

Karl-Heinz Storbeck

Constantine Stratakis

Martha Susiarjo

Gabor Szinnai

Yutaka Takahashi

Hesham Tawfeek

Peter Taylor

Anthony Taylor

Curtis Tilves

Wenxin Tong

Joel Topf

Jorma Toppari

Laura Torchen

David Torpy

Leonardo Trasande

Elena Tsourdi

Adina Turcu

Alfredo Ulloa-Aguirre

Mehmet Uzumcu

Hanneke van Santen

Eszter Vanky

Surendra Varma

Almudena Veiga-Lopez

Joseph Verbalis

Alaina Vidmar

Varsha Vimalananda

Maria Vogiatzi

Frederick Vom Saal

Milka Vrecl

Ana Wägner

Tracy Wang

Leanne Ward

Kate Warde

Emma Watts

Thomas Wehr

Ram Weiss

Robert Wermers

Sarah Wherry

Luiz Eduardo Wildemberg

Karen Winer

Brian Wojeck

Tao-Yiao Wu

Tongzhi Wu

Carol Wysham

Michael Xing

Catherine Yeckel

Saishu Yoshida

Guido Zavatta

Yi Zheng

